# Efficient Array-Based Identification of Novel Cardiac Genes through Differentiation of Mouse ESCs

**DOI:** 10.1371/journal.pone.0002176

**Published:** 2008-05-14

**Authors:** Ronald A. Miller, Nicolas Christoforou, Jonathan Pevsner, Andrew S. McCallion, John D. Gearhart

**Affiliations:** 1 Institute for Cell Engineering, Johns Hopkins University School of Medicine, Baltimore, Maryland, United States of America; 2 McKusick-Nathans Institute of Genetic Medicine, Johns Hopkins University School of Medicine, Baltimore, Maryland, United States of America; 3 Kennedy Krieger Institute, Baltimore, Maryland, United States of America; 4 Department of Obstetrics and Gynecology, Johns Hopkins University School of Medicine, Baltimore, Maryland, United States of America; 5 Department of Molecular and Comparative Pathobiology, Johns Hopkins University School of Medicine, Baltimore, Maryland, United States of America; Lehigh University, United States of America

## Abstract

Remarkably, although cardiac disease accounts for the largest proportion of adult mortality and morbidity in the industrialized world, the genetic programs controlling early cardiogenesis are largely incompletely understood. To better understand this process, we set out to identify genes whose expression is enriched within early cardiac fated populations, obtaining the transcriptional signatures of mouse embryonic stem cells (mESCs) at defined intervals during their differentiation along a cardiac path. We compared the RNA profiles of cardiac precursors cells (CPCs) with time-matched non-CPCs and undifferentiated mESCs, using a transgenic mESC line harboring an *Nkx2-5* cardiac-specific regulatory sequence driving green fluorescent protein (GFP) to facilitate selection of CPCs. We identify 176 transcripts that are significantly elevated in their abundance within CPCs compared with other assayed populations, predicting that they will likely play a role in cardiogenesis. Of note, approximately 24% (43/176) of the cardiogenic candidate transcripts have known roles in cardiac function or development. Importantly, we evaluated the biological relevance of a significant subset 31/133 (23%) of the remaining candidate genes by *in situ* hybridization at multiple time points during development (embryonic day, E7.5–9.5) and report that all were expressed in key cardiac structures during cardiogenesis. Furthermore 9/31, of which many were previously uncharacterized, were detected as early as the formation of the cardiac crescent. These data demonstrate the potential power of integrating genomic approaches with mESC differentiation to illuminate developmental processes, and provides a valuable resource that may be mined to further elucidate the genetic programs underlying cardiogenesis.

## Introduction

The heart is the first organ to form and function in the vertebrate embryo [1]. It is derived from the mesoderm that arises from the primitive streak during gastrulation. In mouse, cardiac progenitors migrate from the middle and anterior region of the streak to a region under the head folds on either side of the midline by E6.5 [2]. At approximately E7.5, these progenitor cells extend across the midline forming what is termed the cardiac crescent, the first anatomically distinct cardiac structure in the developing embryo. The cells of the crescent migrate ventrally to form a linear heart tube consisting of an outer myocardial layer and an inner endocardial layer. Over the next 48 hours, this heart tube undergoes progressing rightward looping, giving rise to a four chambered heart by E10.5.

The developing heart was recently demonstrated to comprise two distinct fields identified through the expression of unique markers, and each giving rise to specific cardiac structures [3], [4]. The secondary heart field, which contributes to the right ventricle and outflow tract, is distinguished by *Hand2*, *Isl1*, and *Fgf10* expression [3]–[5]. Another key cardiac gene, *Nkx2-5,* is simultaneously expressed in both the primary and secondary heart fields, making it a very useful marker for cardiac fated cells during development. *Nkx2-5* is a homeodomain transcription factor and an ortholog of the *Drosophilia* gene *tinman,* a gene that is essential for cardiac specification in the fly [6]. In mouse, *Nkx2-5* appears dispensable for specification and formation of the cardiac crescent. However, it is essential for stages beyond heart tube formation, with embryos lacking *Nkx2-5* exhibiting looping defects and dying *in utero* (E9.5–E11.5) [7]. Although recent studies have provided further insight to mammalian heart development, our understanding of this complicated process is incomplete; elucidation of these processes has been greatly hampered by technical difficulties in studying early mammalian development *in utero*.

We, and others, have attempted to overcome this complication by utilizing the differentiation of embryonic stem cells (ESCs) as a model to study early developmental processes *in vitro.* The expression patterns of several key cardiac genes during ESC differentiation is known to closely reflect their endogenous expression during cardiogenesis *in vivo* [8], [9]. The differentiation of ESCs toward cardiac populations that include cardiomyocytes are characterized by a temporally and spatially ordered expression of specific genes. *Brachyury* expression which specifies mesoderm is followed by *Gata4* and *Nkx2-5* (required for cardiac lineage development), leading to the expression of *αMhc*, *βMhc* and *Cx43* which mark distinct maturation stages [10], and ultimately generating myofibrils and sarcomeres characteristic of mature cardiomyocytes [9]. Although several studies have identified multipotent cardiac progenitor cells (CPCs) in ES-derived populations [11]–[14], we have recently demonstrated that such CPCs are capable of forming all cardiac lineages, including endothelial and vascular smooth muscle in addition to cardiomyocytes [11]–[14]. Although these studies verify the cardiac potential of ESCs most have been performed using multi-lineage populations of cells in which the cardiac lineage represents a minor population.

We set out to establish whether ESCs can serve as a model to provide genetic information on cardiogenesis, determining and comparing transcriptional profiles at different stages during differentiation. To this end we have isolated GFP positive (GFP^+^) and GFP^−^ populations at three developmentally significant time points during their differentiation, simultaneously separating GFP^+^ and GFP^−^ populations within embryoid bodies. Our first time point was established as the undifferentiated starting population and was designated day 0 (D0). At this time our mESCs express markers of pluripotency such as *Oct4* and *SSEA-1* [11]. As differentiation proceeds, these mESCs decrease expression of the pluripotency markers and by the fourth day of the differentiation process (D4) these mESCs this cell population expresses the mesodermal marker *Brachyury* [11]. This was selected as our second time point. The third and final time point utilized in this study, day 6 (D6), is characterized by the initiation of robust *Nkx2-5* expression, a critical gene expressed in the earliest stages of cardiac development, the formation of the cardiac crescent. This pivotal cardiac marker is expressed within a substantial subset of the differentiating population, but not in all cells and it remains expressed for the life of the cardiomyocyte *in vitro* and *in vivo*. Importantly expression of the GFP reporter occurs in concert with *Nkx2-5* expression in the cardiac fated cell population, making it possible to obtain a discrete population of cardiac *Nkx2-5* expressing by fluorescence activated cell sorting (FACS). RNA isolated from these populations was then subjected to array-based transcript profiling analyses. We compared the expression profile of the GFP^+^ population with earlier time points and time matched non-cardiac fated cells (GFP^−^).

We predicted that transcripts upregulated in the GFP^+^ population relative to earlier time points or non-cardiac fated cells, would most likely be genes expressed in cardiovascular structures during cardiac development. Of the genes upregulated in the GFP^+^ population, 43/176 (24%) have known roles in cardiac or hematopoietic development or function. The significant remainder (133/176) consists of genes not previously known to function in cardiogenesis. Indeed, more than 20% (52/260) of the transcripts differentially regulated in the GFP^+^ population have no known function. We then asked what which genes lacking an identified role in cardiac development were appropriately expressed in cardiac structures. Through the assessment of 31 candidate genes at 3 critical points during heart development: E7.5, the cardiac crescent; E8.5, the heart tube; and E9.5, the looping heart; we demonstrated that all were expressed as predicted within appropriate cardiovascular structures, including 9/31 (29%) that were expressed in the cardiac crescent or heart tube at the earliest stages of heart formation. These data validate the power of ESC differentiation strategies as a model for mammalian development and, in combination with established genomic technologies, as a powerful gene discovery tool for developmental processes.

## Methods

### Cell Culture and Differentiation Strategy

mESCs were differentiated through embryoid body [15] formation (hanging droplet technique) as described [16]. Following primary mouse embryonic fibroblasts (PMEF) feeder subtraction, mESCs were dissociated and resuspended in differentiation medium at a density of 5×104 cells/mL. Cells at this time point were designated as day 0 (D0). Following incubation for two days, the EBs were transferred, in suspension, to poly-HEMA coated tissue culture dishes to prevent cell attachment and grown in medium containing ascorbic acid [17] which was replenished every 3 days thereafter. The generation of the mESC line used in these experiments has been previously described [11] and harbors a 6.7 kb Nkx2-5 cardiac specific enhancer sequence [15] driving GFP.

### Fluorescence Activated Cell Sorting

EBs were harvested on days 4 and 6 (D4 and D6), washed in PBS for 30 minutes at 37°C then resuspended in 0.25% trypsin for 4, and 7 minutes, respectively. Cells from each time point (D0, D4, and D6) were sorted using the BD Biosciences FACSAria™ Cell sorting system. D6 cells alone were sorted into GFP+ and GFP− fractions; a small fraction of these cells were collected in PBS for post-sort analysis, the remainders were sorted into RLT buffer (RNeasy, Qiagen) as were the D0 and D4. Data collection for each time point was performed in triplicate.

### RNA Preparation and Array Hybridization

The isolated RNA was amplified and labeled using the Affymetrix Two Cycle™ labeling kit according to protocols described by the manufacturer. The resulting labeled cRNA was hybridized to Affymetrix mouse genome GeneChip 430 arrays, version 2.0. Fluorescence was detected using the Affymetrix-GS3000 GeneArray™ Scanner and image analysis of each GeneChip™ was performed using the GeneChip™ Operating System software from Affymetrix.

### Data Analysis

Microarray data were preprocessed (background subtraction, normalization, and summarization) using robust multi-chip analysis (RMA) [18]. A linear mixed-model ANOVA was used to detect differential transcript expression based on the time point and presence or absence of a GFP population (GFP positive populations were cells fated along a cardiac lineage). A comparison was made among the Day 6 GFP+ population and the Day 0, Day 4, and Day 6 GFP- population. All analyses were performed using the Partek® Genomic Suite software package version 6.3 Beta.

### Quantitative RT-PCR

RNA was reverse transcribed using random decamers and MMLVRT. RNA levels were measured using FAM labeled probes from Applied Biosystems: Ramp2 (Mm00490256_g1), Nkx2-5 (Mm00657783_m1), Tek (Mm00443242_m1), Gyg1 (Mm00516516_m1), Myla (Mm00440378_m1), Gata4 (Mm00484689_m1), and Isl1 (Mm00627860_m1); all probes were normalized to 18S (Hs99999901_s1). Samples were collected in an Applied Biosystems ABIPrism 7900HT and analyzed using SDS2.1 software.

### Gene Ontology Annotation

The ANOVA filtered Affymetrix Mouse 430_2.0 probe sets were assigned to functional categories based largely on Gene Ontology Consortium (GO) annotation [19], (http://geneontology.org). The protein-based GO annotation terms were mapped to genes by the Mouse Genome Database (MDG) of Mouse Genome Informatics [20], http://www.informatics.jax.org/. Affymetrix expression probes were mapped to a transcript in their NetAffx Annotation files, which also include a subset of the genes' GO annotations. To supplement the Affymetrix GO annotation this analysis also employed the Spotfire® (www.spotfire.com) Functional Genomics platform to access full GO annotation.

### Nucleic Acid in situ Hybridization

Probes 1–11 of the candidate genes were generated from E15 whole mouse RNA. A ∼600 bp region from PCR amplified from the cDNA and TA cloned into the Invitrogen PCRII vector (Primers available upon request). Probes 12–31 were obtained through Invitrogen Clone Ranger ©. Nkx2-5 probe was a gift from Dr. Richard P. Harvey [21]; Isl1 probe was a gift from Dr. Sylvia Evans [5]. Digoxygenin-labelled riboprobes were synthesized using Sp6, T3, and T7 RNA Polymerase. CD-1 embryos were collected on days E7.75, E8.5, and E9.5, and in situ hybridization was performed as previously described [22].

## Results

### Isolation of ESCs Differentiating Along a Cardiac Lineage

**Figure 1:**
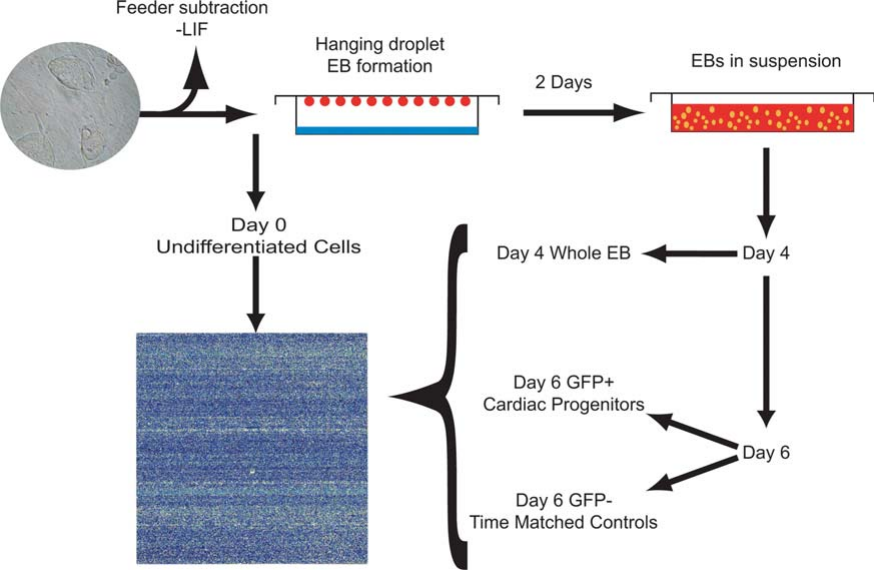
Description Of Experimental Design. mESCs were differentiated within hanging droplets and RNA was isolated from the corresponding undifferentiated and differentiating mESC populations at specific time points (day 0, day 4, day 6 GFP^+^, and day 6 GFP^−^; Methods). Isolated RNA populations were then hybridized to Affymetrix 430 2.0 microarrays and the resulting transcription profiles analyzed as described in Methods.

In an effort to give access to cardiac fated populations at the earliest stages of development, we have made use of a transgenic mESC line (*Nkx2-5-GFP*) containing an established cardiac specific enhancer element of the *Nkx2-5* transcription factor [15] driving expression of the green fluorescent protein (Methods). This cell line was subjected to cardiac differentiation strategies described in the methods; we selected specific time points for analysis at which transcriptional changes in key pluripotent/mesodermal/cardiac genes indicate critical stages in cardiogenesis: D0 representing undifferentiated mESCs; D4 corresponding to the differentiation along a mesodermal lineage; and D6, with the onset of robust *Nkx2-5* expression in the cardiac progenitor population. Cells were harvested at all selected time points and FAC sorted, with only the day 6 samples were sorted into GFP^+^ and GFP^−^ fractions (36.9%±9.8% of the cells expressed GFP in the day 6 indicating a high level of cardiac enrichment). In order to assess the efficiency of our FAC sorting to discriminate between the GFP^+^ and GFP^−^ population, we performed an additional round of flow cytometry, which demonstrated a high purity of the GFP^+^ and GFP^−^ populations, 95.7%±3.4% and 99.5%±1.5% respectively (Figure S1). We isolated RNA from undifferentiated (D0), mixed (cardiac fated and non-cardiac fated; D4), and FAC sorted D6 non-cardiac fated (GFP negative; GFP^−^) and cardiac fated (GFP positive; GFP^+^) cell populations (Figure 1); RNA isolations were performed on independent cell isolates of each population in triplicate (Methods)

### Analyses of Transcriptional Profiles and Identification of Cardiac Candidate Genes

We examined RNA from four groups (day 0; day 4; day 6 GFP^+^; day 6 GFP^−^; n = 3 samples per group). RNA was isolated from the cell populations, amplified, labeled and hybridized to Affymetrix Genechip 430 version 2.0 arrays as described in Methods. Expression data were normalized using Robust Multichip Average (RMA), consisting of three steps: a background adjustment, quantile normalization and finally summarization [18]. In order to determine the integrity of the data generated in our array experiment, we undertook several analytical approaches (PCA, hierarchical clustering, and *p*-value analysis), which are established strategies to evaluate microarray data.

**Figure 2:**
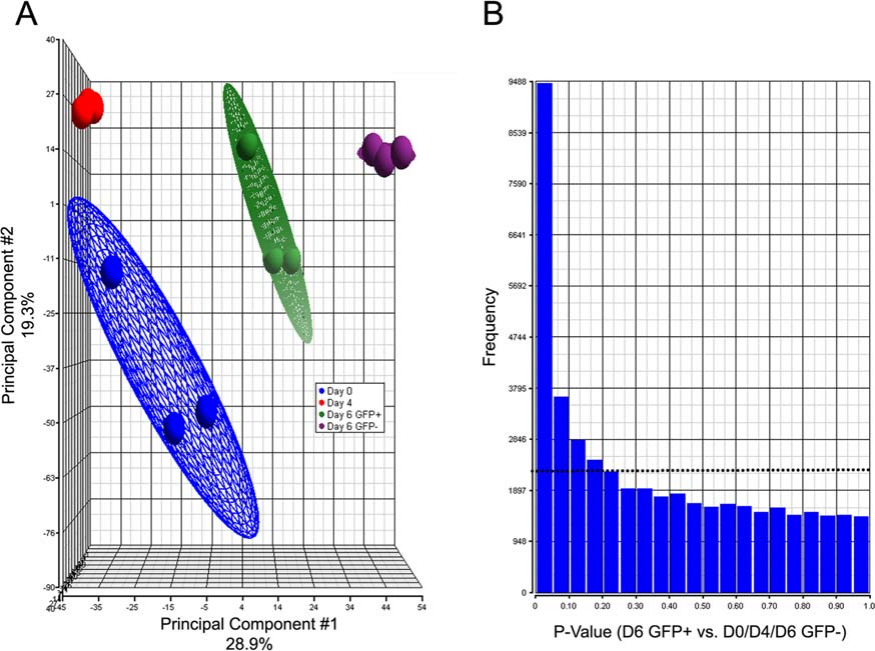
Determination Of Data Integrity. A. Principle components analysis (PCA) of the four experimental groups: day 0, day 4, day 6 GFP^+^, and day 6 GFP^−^. B. Distribution of observed *p*-values. The dotted line corresponds to the frequency of *p*-values if they were uniformly distributed across all bins.

Principal components analysis (PCA) is an unsupervised technique in which the data are not segregated into groups prior to the analysis. Instead, patterns are observed based on the output of the analysis. To this end we used Partek software with default settings, including a covariance dispersion matrix (Methods). The data used to generate this plot consist of a matrix of 12 samples, each comprising 45,101 RNA transcript intensity values. The axes correspond to the principal components (PCs), with the first PC axis accounting for 28.9% of the variance and the second PC axis accounting for 19.3% of the variance. The plot showed the samples in four groups corresponding to the four experimental conditions. This demonstrates that the samples were distinguishable based on their overall gene expression profiles, that the samples were normalized consistently, and that there were no outlier samples (Figure 2A). Our ability to discriminate between experimental groups by PCA was consistent with hierarchical clustering analyses (data not shown). These analyses gave us confidence in the integrity of our data and we therefore proceeded to test the hypothesis that day 6 GFP^+^ cells (which represented the cardiac progenitor population) possess a unique transcriptional signature relative to control populations (D0, D4 and D6 GFP^−^). To test this hypothesis we implemented ANOVA using a “contrast” of the day 6 GFP^+^ versus the “control” populations (Methods). We applied several false discovery rates (0.05, 0.01, 0.001), identifying 2643 (FDR = 0.05), 1018 (FDR = 0.01) and 297 significantly regulated probe sets, respectively (FDR = 0.001; Table 1, Table S1). We also demonstrated that a large proportion of transcripts were significantly regulated in the day 6 GFP^+^ cells by plotting a histogram of the frequency of *p*-values (y-axis) versus the observed *p*-value (x-axis) (Figure 2B). If our comparisons of contrast (GFP^+^) RNAs to controls (D0, D4 and D6 GFP^−^) were to demonstrate no significant differential regulation, we would expect a random distribution of *p*-values resulting in 2255 *p*-values in each bin. Our data demonstrated that the observed number of small p-values is clearly overrepresented, an indication of robust power to discriminate between the groups being compared.

**Table 1:** The Numbers of Transcripts Whose Levels Vary Significantly Between Conditions

FDR level	*p*-value	RNA Transcripts Identified
0.001	6.58×10^−6^	297
0.01	2.26×10^−4^	1018
0.05	2.93×10^−3^	2643

RNA Transcripts Identified corresponds to the number of transcripts present at each significance threshold comparison of day 6 GFP^+^ cells to controls (day 0, day 4, and day 6 GFP^−^). FDR, false discovery rate; *p*-value, significance threshold determined for each FDR.

**Figure 3:**
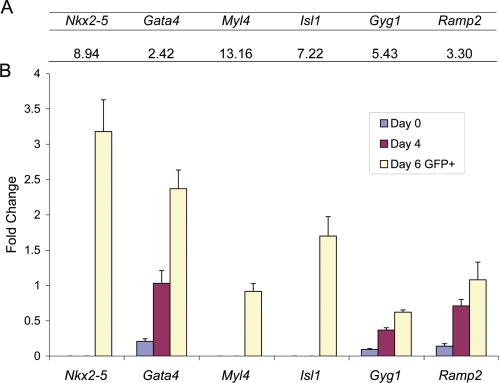
Confirmation of Microarray Data by qRT-PCR. A. Geometric fold change values of the selected genes (GENE names) in the contrast of Day 6 GFP^+^ versus day 0, day 4 and day 6 GFP^−^. B. qRT-PCR of the selected genes at day 0, day 4 and day 6 GFP^+^ time points. Error bars represent the normalized standard deviation.

To establish the integrity of the array-based data and these statistical analyses we performed qRT-PCR on a subset of known cardiac genes (*Nkx2-5*, *Gata4*, *Isl1*), as well as genes shown by Masino et al., (2004) to be upregulated in developing cardiomyocytes evaluated *ex vivo* [23] (*Tek, Gyg)* (Figure 3). All qRT-PCR data were consistent both with the published data and with the enrichment of these transcripts in the D6 GFP^+^ population relative to the D0 and D4 populations detected within the array experiment. These data further validated the statistical analysis of our array data and support its potential *in vivo* biological relevance. Thus we moved on to impute some biological correlates to this large body of data.

### GO Annotation of Differentially Regulated Transcripts

**Figure 4:**
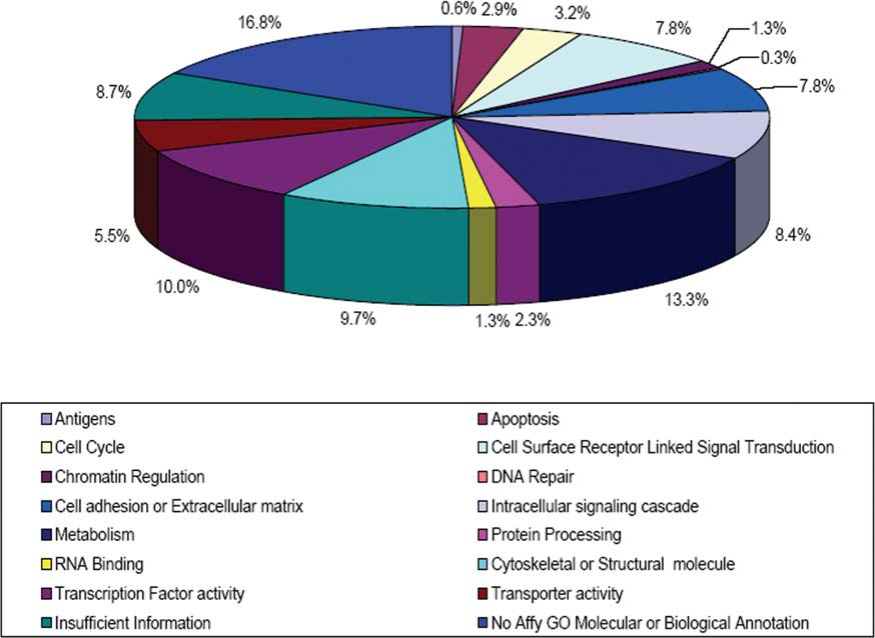
Gene Ontology of Dataset Transcripts. Pie chart representing the relative abundance of transcripts within the dataset of genes displaying significantly different levels in the contrast of Day 6 GFP^+^ versus day 0, day 4 and day 6 GFP^−^. Transcripts were classified according to Gene Ontology (GO) terms. Annotated transcripts whose ontology could not be determined based on available information were classified as having “insufficient information”. Transcripts with no annotation were also reported as “no annotation”.

To determine the function of the transcripts upregulated in the Day 6 GFP^+^ population, we queried ontology databases. At FDR ≤0.001, the identified 297 probe sets represented 260 unique genes or transcripts, of which approximately 80% (208/260) had GO annotation. The corresponding GO annotations were established for processes, functions, or cellular locations that have been related to cardiac differentiation and the results presented in Table S2. The one exception is that “antigens”, which is not a GO classification, were selected by their MGD Nomenclature Committee names. The resulting ontology data indicates that there were significant fractions of genes, upregulated in the D6 GFP^+^ population, responsible for cell signaling (extracellular, 24/260, 9%; and intracellular, 26/260, 10%) as well as transcriptional regulation (31/260, 12%). There is also a large population of structural molecules (30/260, 12%) in this dataset, including myosins and troponins, indicative of a cardiogenic population. Finally the largest population of the ontology dataset is transcripts of unknown function (52/260; 20%), and an additional 10% (27/260) of the identified transcripts providing insufficient information for definite classification (Figure 4). Although these data suggest trends in biological classification of identified genes, they fall short of determining their biological relevance.

To this end we set out to define the developmental expression of selected candidates, we selected those transcripts that were upregulated in GFP^+^ population (n = 176) choosing as a threshold a high stringency FDR of 0.001. In addition, we selected only those transcripts displaying a minimum fold change threshold of +1.2. Importantly, of 176 transcripts upregulated in the cardiac D6 population, 43 (24%) have known roles in cardiac development or function (Table S1). Our underlying hypothesis was straightforward; identified genes highly likely to be expressed in key cardiac structures during development.

### Determination of Biological Significance in vivo by in situ

**Figure 5:**
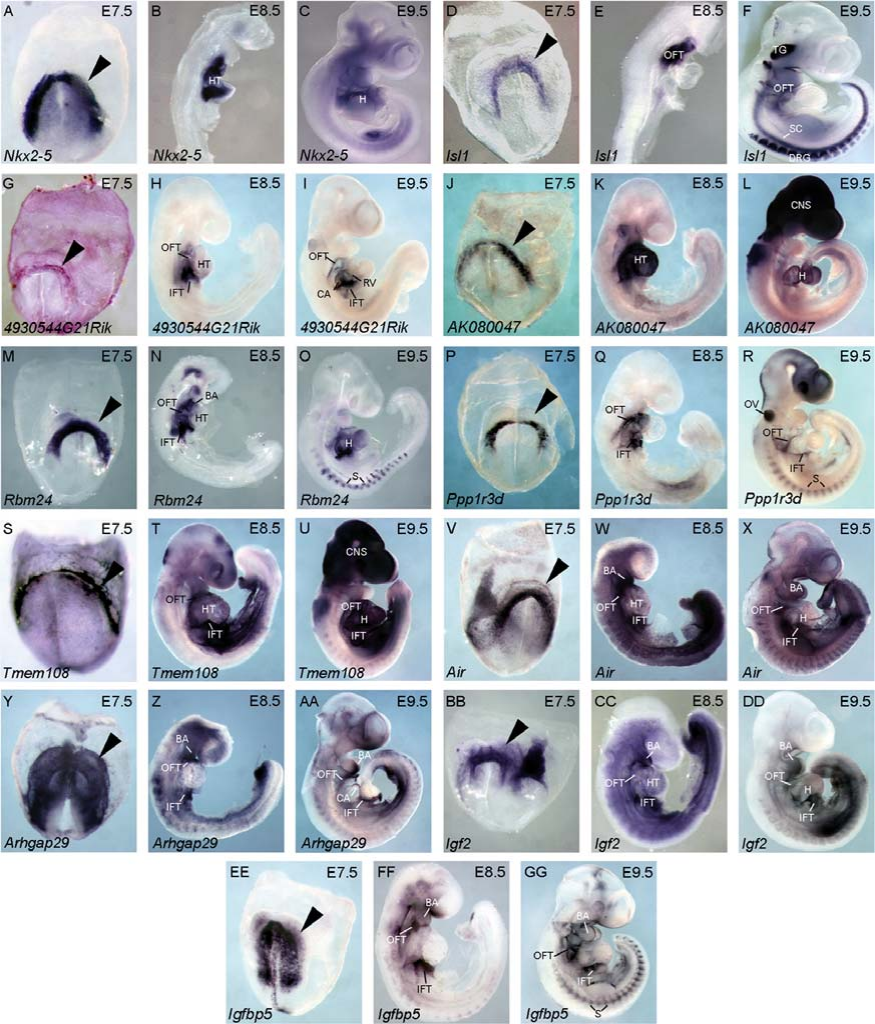
Many Identified Candidates are Detected in the Cardiac Crescent. Embryonic expression of transcripts was evaluated in mice at E7.5, E8.5 and E9.5. *Nkx2-5* (A-C) and *Isl1* (D-F) were used as anatomical controls for cardiac expression. *4930544G21Rik* (G-I), *AK080047* (J-L), *Rbm24* (M-O), *Ppp1r3d* (P-R), *Tmem108* (S-U), *Air* (V-X), *Arhgap29* (Y-AA), *Igf2* (BB-DD), *Igfbp5* (EE-GG) were all expressed in the cardiac crescent of E7.5 embryos (arrowheads). HT, heart tube; H, heart; OFT, out flow tract; IFT, inflow tract; CA, common atria; RV, right ventricle; CNS, central nervous system; BA, branchial arches; S, somites; OV otic vesicle; SC, spinal cord; DRG, dorsal root ganglia; E7.5-9.5; embryonic days 7.5-9.5.

We selected 31 candidates of the remaining 133 for evaluation (23%), prioritizing genes for which insufficient biological data were available to include or exclude a role in cardiogenesis. We determined the spatial expression of selected candidates by *in situ* hybridization at mouse E7.5, E8.5, and E9.5, critical time points for mouse embryonic heart development. To aid our analysis, we first established the expression patterns of two known cardiac genes; *in situ* hybridization was performed using probes for *Nkx2-5* and *Isl1* at the corresponding time points (Figure 5 A–F). The transcript corresponding to the *Nkx2-5* transcription factor is present in the primary and secondary heart fields at E7.5, the linear heart tube at E8.5, and throughout the looping heart at E9.5 (Figure 5 A–C). Secondly, *Isl1* is expressed in the secondary (anterior) heart field at E7.5; its expression persists in the outflow tract at E8.5; and subsequently in the outflow tract, trigeminal ganglia, spinal cord, and dorsal root ganglia on E9.5 (Figure 5 D–F).

The candidates evaluated by *in situ* hybridization demonstrated a wide range of expression levels and locations throughout the studied time points. Consistent with our hypothesis that the candidates upregulated in the GFP^+^ population are expressed in early cardiac structures, transcripts corresponding to nine of the genes (*4930544G21Rik, Ak080047, Rbm24, Ppp1r3d, Tmem108, Air, Arhgap29, Igf2,* and *Igfbp5*) were detected in the cardiac crescent, at the earliest timepoint studied (E7.5) (Figure 5). Four candidates, *4930544G21Rik, Ak080047, Rbm24,* and *Ppp1r3d,* were expressed exclusively in the cardiac crescent (Figure 5 G, J, M, P); while *Tmem108, Air, Arhgap29, Igf2,* and *Igfbp5* were expressed in the cardiac crescent and in other early embryonic structures (Figure 5 S, V, Y, BB, EE). At E8.5, the cell populations in which these genes were expressed correspond to discrete cardiac structures, with *Ak080047* expressed solely in the heart tube (Figure 5K); *4930544G21Rik, Tmem108,* and *Rbm24* expressed in the heart inflow tract, heart tube and outflow tract (Figure 5 H, N, T); *Ppp1r3d* expressed in the inflow and outflow tracts (Figure 5Q); *Arhgap29* and *Igfbp5* expressed in the branchial arches, inflow and outflow tract (Figure 5 Z, FF); and *Air* and *Igf2* expressed in the inflow tract, heart tube, and outflow tract, in addition to other embryonic tissues (Figure 5 W, CC). At E9.5, *4930544G21Rik* was expressed in the inflow tract, common atria, right ventricle, outflow tract, and central nervous system (CNS) (Figure 5I); whereas *Ak080047* was expressed throughout the heart and CNS (Figure 5L); similarly *Rbm24* was expressed in the entire heart but is also detectible in the somites (Figure 5O). *Arhgap29* was expressed in the inflow tract, common atria, outflow tract, and branchial arches and other embryonic tissues (Figure 5AA); while *Ppp1r3d* was inflow and outflow tracts, otic vesicle, and somites (Figure 5R); *Tmem108* was expressed in the inflow tract, heart, outflow tract, and CNS (Figure 5U); *Igfbp5* was expressed in the inflow and outflow tracts, branchial arches, and somites (Figure GG); finally *Igf2* and *Air* were expressed in the inflow tract, heart, outflow tract, and branchial arches along with other embryonic tissues (Figure 5 X, DD).

**Figure 6:**
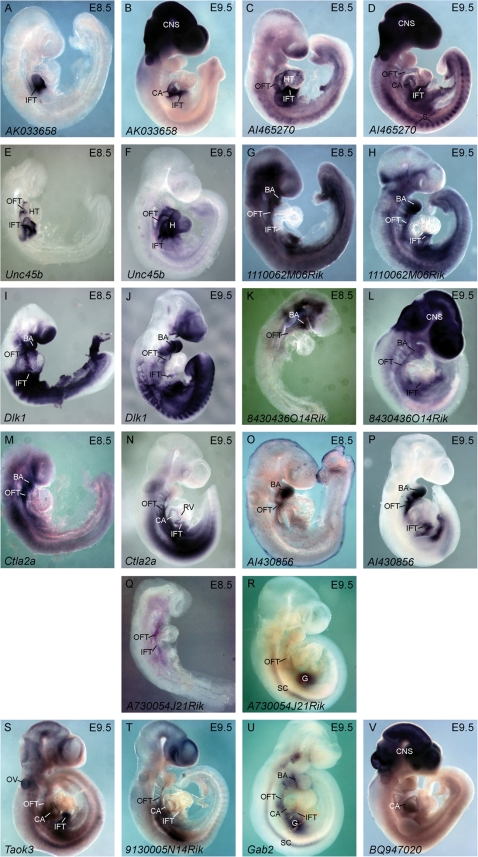
Candidate Genes are Frequently Expressed in Cardiac Structures. *AK033658* (A–B), *AI465270* (C–D), *Unc45b* (E–F), *1110062M06Rik* (G–H), *Dlk1* (I–J), *8430436O14Rik* (K–L), *Ctla2a* (M–N), *AI430856* (O–P), and *A730054J21Rik* (Q–R) detected in cardiac structures at E8.5 and E9.5. *Taok3* (S), *9130005N14Rik* (T), *Gab2* (U), and *BQ947020* (V) detected in cardiac structures at E9.5. HT, heart tube; H, heart; OFT, out flow tract; IFT, inflow tract; CA, common atria; RV, right ventricle; CNS, central nervous system; BA, branchial arches; S, somites; OV otic vesicle; SC, spinal cord; G, gut.

Although transcripts corresponding to the remaining candidates could not be detected at E7.5, many were detectable by E8.5. *AK033658* was expressed in the inflow tract of the E8.5 and E9.5 embryo, with expression also detected in the common atria and CNS of the E9.5 embryo (Figure 6 A–B). *AI465270* and *Unc45b* were expressed in the inflow tract, heart, and outflow tract of the E8.5 and E9.5 embryo (Figure 6 C–F). Other candidate transcripts (*Dlk1*, *1110062M06Rik*, and *8430436O14Rik*) were detected in key cardiac structures such as the branchial arches, inflow tract and outflow tract, but not the heart itself (Figure 6 G–L). *Ctla2a* is expressed in the branchial arches and outflow tract of the E8.5 embryo, with expression later seen in the inflow tract, common atria, right ventricle and outflow tract of the E9.5 embryo (Figure 6 M–N). *AI430856* was expressed in the branchial arches and outflow tract of the E8.5 mouse embryo (Figure 6O); in the E9.5 embryo, expression was observed in the branchial arches, inflow tract, and outflow tract (Figure 6P). *AI430856* was later identified to be a portion of the 3′ untranslated region (UTR) of the *Hand2* gene, but the absence of expression in the right ventricle, in contrast to the *Hand2* gene [24], indicates a possible regulatory role of this fragment. Finally, *A730054J21Rik* was expressed in the inflow and outflow tract at E8.5 (Figure 6Q); at E9.5, *A730054J21Rik* expression continued in the outflow tract, and was also shown to be expressed in the spinal cord and a position consistent with the forming gut (Figure 6R).

Finally, the expression of some candidates was only detectible by E9.5, with *Taok3* expression observed in the inflow tract, common atria outflow tract, and otic vesicle of the E9.5 embryo (Figure 6S); *9130005N14Rik* expression observed in the inflow tract, common atria, and outflow tract at E9.5 (Figure 6T); expression of *Gab2* evident in the branchial arches, inflow tract, common atria, spinal cord, and forming gut of E9.5 embryos (Figure 6U); and *BQ947020* was expressed in the right ventricle and CNS of the E9.5 embryo (Figure 6V). The remaining eight candidates evaluated through *in situ* hybridization were expressed in cardiac related structures such as the heart, outflow tract, and branchial arches, as well as other embryonic structures (Figure S2).

A table summarizing the results of 31 *in situ* hybridizations completed in this study provides a listing of developmentally important cardiac structures in which they are expressed (Table 2). Our data demonstrate the successful utilization of mESCs to model and illuminate early cardiac development. By acquiring a transcriptional profile of these cells at multiple times during their differentiation along a cardiac lineage, we have been able to identify both known and novel transcripts expressed in early cardiac structures *in vivo*, providing a repository of genes with potential functional significance in cardiogenesis.

**Table 2:** Summary of sites of Transcript Expression Detected by *in situ* Hybridization

Probeset ID	Gene Symbol	E7.5	E8.5	E9.5
1455040_s_at	*1110062M06Rik*	No Signal	BA, IFT, OFT†	BA, IFT, OFT†
1417877_at	*2310005P05Rik*	No Signal	No Signal	BA, IFT, H, OFT †
1435399_at	*2310068J10Rik*	No Signal	No Signal	IFT, CA, OFT, CNS †
1454926_at	*4930544G21Rik*	Crescent	IFT, HT, OFT	IFT, CA, RV, OFT
1452762_at	*8430436O14Rik*	No Signal	BA, OFT	BA, IFT, OFT, CNS
1452761_a_at	*8430436O14Rik*	No Signal	BA, OFT	BA, IFT, OFT, CNS
1417272_at	*9130005N14Rik*	No Signal	No Signal	IFT, CA, OFT
1438531_at	*A730054J21Rik*	No Signal	IFT, OFT	OFT, SC, G
1437401_at	*AI425372*	No Signal	No Signal	BA, IFT, CA, OFT, CNS †
1436041_at	*AI430856*	No Signal	BA, OFT	BA, IFT, OFT
1438705_at	*AI465270*	No Signal	IFT, HT, OFT	IFT, CA, OFT, S
1456139_at	*Air*	Crescent †	BA, IFT, HT, OFT †	BA, IFT, H, OFT †
1435265_at	*AK033658*	No Signal	IFT	IFT, CA, CNS
1434766_at	*AK080047*	Crescent	HT	H, CNS
1454745_at	*Arhgap29*	Crescent †	BA, IFT, OFT †	BA, IFT, CA, OFT †
1434413_at	*BG092677*	No Signal	No Signal	IFT, CA, OFT †
1428891_at	*BI455251*	No Signal	DRG	BA, CA, OFT, DRG †
1437967_at	*BQ947020*	No Signal	No Signal	CA, CNS
1416811_s_at	*Ctla2a /// Ctla2b*	No Signal	BA, OFT †	IFT, CA, RV, OFT †
1452352_at	*Ctla2b*	No Signal	BA, OFT †	IFT, CA, RV, OFT †
1449939_s_at	*Dlk1*	No Signal	BA, IFT, OFT †	BA, IFT, OFT †
1419829_a_at	*Gab2*	No Signal	No Signal	BA, IFT, CA, OFT, G, SC
1440830_at	*Gpr116*	No Signal	No Signal	BA, IFT, CA, OFT †
1418379_s_at	*Gpr124*	No Signal	No Signal	BA, IFT, OFT †
1448152_at	*Igf2*	Crescent †	BA, IFT, HT, OFT †	BA, IFT, H, OFT†
1452114_s_at	*Igfbp5*	Crescent †	BA, IFT, OFT †	BA, IFT, OFT, S †
1452922_at	*Ppp1r3d*	Crescent	IFT, OFT	IFT, OFT, OV, S
1456180_at	*Rbm24*	Crescent	BA, IFT, HT, OFT	H, S
1458624_at	*Rbm24*	Crescent	BA, IFT, HT, OFT	H, S
1435964_a_at	*Taok3*	No Signal	No Signal	IFT, CA, OFT, OV
1423250_a_at	*Tgfb2*	No Signal	BA, IFT, OFT †	BA, IFT, OFT †
1436916_at	*Tmem108*	Crescent †	IFT, HT, OFT	IFT, H, OFT, CNS
1454729_at	*Tmem108*	Crescent †	IFT, HT, OFT	IFT, H, OFT, CNS
1436939_at	*Unc45b*	No Signal	IFT, HT, OFT	IFT, H, OFT
1423835_at	*Zfp503*	No Signal	BA, OFT †	BA, OFT †

Sites of expression are tabulated for each of 31 candidates evaluated at each of 3 embryonic stages during cardiogenesis. HT, heart tube; H, heart; OFT, out flow tract; IFT, inflow tract; CA, common atria; RV, right ventricle; CNS, central nervous system; BA, branchial arches; S, somites; OV otic vesicle; SC, spinal cord; G, gut; DRG, dorsal root ganglia; E7.5–9.5; embryonic days 7.5–9.5;

† indicates expression of transcript was detected in many other structures throughout the embryo. No signal means that expression was not detected at that time point.

## Discussion

Elucidating the genetic programs underlying developmental processes is a central goal in contemporary medical research. Small populations of target cells that are not easily isolated from the surrounding tissue can complicate this challenge. We demonstrate that *in vitro* differentiation strategies using mESCs can effectively model the early stages of cardiogenesis, and that established genomic technologies can be effectively used to illuminate pertinent genetic signatures therein. Through the use of the *Nkx2-5-*GFP ES cell line we have isolated a relatively pure cardiac progenitor population for transcriptional profiling. By comparing the cardiac progenitor population (D6 GFP^+^) against undifferentiated mESCs (D0), early differentiating mESCs (D4) and non-cardiac multi-lineage mESCs (D6 GFP^−^), we generated a robust dataset represented by 260 unique transcripts significantly differentially expressed (FDR of 0.001). We have validated the integrity of this data through qRT-PCR and demonstrated its concordance with a sample of genes identified in a previous *ex vivo* analysis (Masino et al., 2004). Consistent with the underlying premise of this experiment, 43/176 (24%) of the transcripts upregulated in the cardiac fated population were known to play a role in cardiogenesis or hematopoiesis, confirming the strength of this dataset. While a significant fraction of the transcripts in this dataset were of unknown function (52/260), we posit that many play a role in cardiogenesis.

Analysis of the gene ontology of differentially expressed transcripts, while imperfect, provides insight to the potential interactions that underlie the fate determination that drives the mesodermal subpopulation of differentiating mESCs along a cardiac lineage. Although the complex signaling pathways necessary for cardiogenesis are incompletely known, the large number of transcripts in this dataset encoding transcription factors (n = 32) or genes involved in extracellular (n = 24) or intracellular (n = 26) signal transduction provide novel avenues for the investigation of cardiac development. While some of the transcription factors identified in this study, such as *Nkx2-5, Tbx5,* and *Mef2c,* have an established role in cardiogenesis, many do not, making them ideal candidates for further appraisal. Finally, the large number of transcripts with no (n = 52) or incomplete (n = 27) annotation offer an equally novel foundation for future work in this field. Importantly, the candidacy of several novel players must first be dependent on a demonstration of their biological relevance to cardiogenesis.

We set out to establish the biological relevance of a large subset of candidates through *in situ* hybridization during embryogenesis and in doing so we uncovered a wide range of expression patterns consistent with cardiogenesis. Most significantly, nine of the 31 candidates (29%) (*4930544G21Rik, Ak080047, Rbm24, Ppp1r3d, Tmem108, Air, Arhgap29, Igf2,* and *Igfbp5*) are expressed in the forming cardiac crescent (E7.5). The expression of these genes is consistent with their potential role in the formation of the earliest cardiac structures. Furthermore, these genes continue to be expressed at subsequent time points (E8.5, E9.5) consistent with a possible role in the maintenance or maturation of cardiac lineages. Although transcripts corresponding to the remaining 22 candidates evaluated in this study were not detected at E7.5, all were shown to be expressed in cardiovascular tissues at later time points (E8.5, E9.5). The expression of these transcripts at later time points does not diminish their potential importance in cardiogenesis; they may also play significant roles in the maturation of the early cardiogenic population, or the morphological changes to the structure of the looping heart.

While many of these candidates are expressed solely in cardiac structures, the expression of most major cardiac genes extends beyond the cardiovascular system. *Nkx2-5,* for example, is also expressed in the foregut, thyroid, spleen, stomach and tongue [25]; similarly, in addition to the outflow tract *Isl1* is expressed in the spinal cord, dorsal root ganglia, and trigeminal ganglia at E9.5; and *Tbx5* is expressed in both the heart as well as the limb bud [26]. Consistent with these observations, many of the candidates evaluated in this study are also expressed in both cardiac and non-cardiac structures.

Importantly *Nkx2-5* is expressed in both the primary and secondary heart fields. Consequently we anticipated that our transcriptional analyses could identify transcripts present in both heart fields, as well as those exclusively present in one heart field. Of the candidate genes investigated, we would predict *Igfbp5, 4930544G21Rik,* and *Ppp1r3d* are expressed in the secondary heart field at E7.5. Consistent with lineage tracing experiments of the secondary heart field [3], [5], these genes are later expressed in the outflow tract at E8.5 and E9.5 and the developing atria and right ventricle at E9.5. Conversely, *AK080047* is expressed in a region consistent with the primary heart field at E7.5, with later expression in the linear heart tube at E8.5 and throughout the heart entire heart at E9.5. These examples underscore the ability of the *Nkx2-5-GFP* cell line to isolate multiple populations of cells that contribute to the developing heart.

Although the candidate genes whose biological relevance has been evaluated in this study represent an incomplete sample of the total dataset, we have shown that many transcripts with no prior functional annotation display expression tight and specific patterns consistent with genes involved in cardiogenesis. While we have not experimentally validated the biological relevance of every transcript that is upregulated in the cardiac fated GFP^+^ population, taken in concert with published data we demonstrate that more than 40% (74/176) had expression patterns consistent with a role in cardiogenesis. First, 24% (43/176) of the genes identified in this screen are known to have an established role in cardiogenesis or hematopoiesis. Second, of the genes upregulated in the cardiac fated population, but lacking an identified role in cardiogenesis (133/176), 31 are detected in early cardiac structures by *in situ* hybridization. Taken collectively, these data strongly support the utility of mESCs to model early cardiac development. These data will serve as a great resource as we continue to investigate the mechanisms of cardiac development; focusing on further exploration of this dataset and characterizing the mechanisms by which these newly identified cardiac genes participate in cardiac development.

## Supporting Information

Table S1 Results of array comparison.
(1.44 MB XLS)

Table S2 Gene ontology tables.
(0.27 MB XLS)

Figure S1 FAC Sorting Yields a Highly Enriched Population of Cardiac Fated Cells. FAC Sorting was performed on day 6 of differentiation, separating the cardiac fated cells (GFP+) from the non-cardiac fated population (GFP-) (A). The resulting samples were subjected to a second round of analyses to determine the purity of the cardiac (B) and non-cardiac (C) populations
(9.95 MB TIF)

Figure S2 Candidate Genes Displaying Widespread Embryonic Expression in Tissues Including Key Cardiac Structures. Tgfb2 (A–B), Zfp503 (C–D), BI455251 (E–F), 2310068J10Rik (G–H) detected in cardiac structures at E8.5 and E9.5. AI425372 (I), BG092677 (J), Gpr116 (K), and Gpr124 (L) detected in cardiac structures at E9.5. H, heart; OFT, out flow tract; IFT, inflow tract; BA, branchial arches; CA, common atria; DRG, dorsal root ganglia; CNS, central nervous system.
(9.93 MB TIF)
