# Supplementary figures and images for: Efficient Array-Based Identification of Novel Cardiac Genes through Differentiation of Mouse ESCs
Source: PLoS One. 2008 May 14;3(5):e2176. doi: 10.1371/journal.pone.0002176 (PMC2364653; doi:10.1371/journal.pone.0002176)

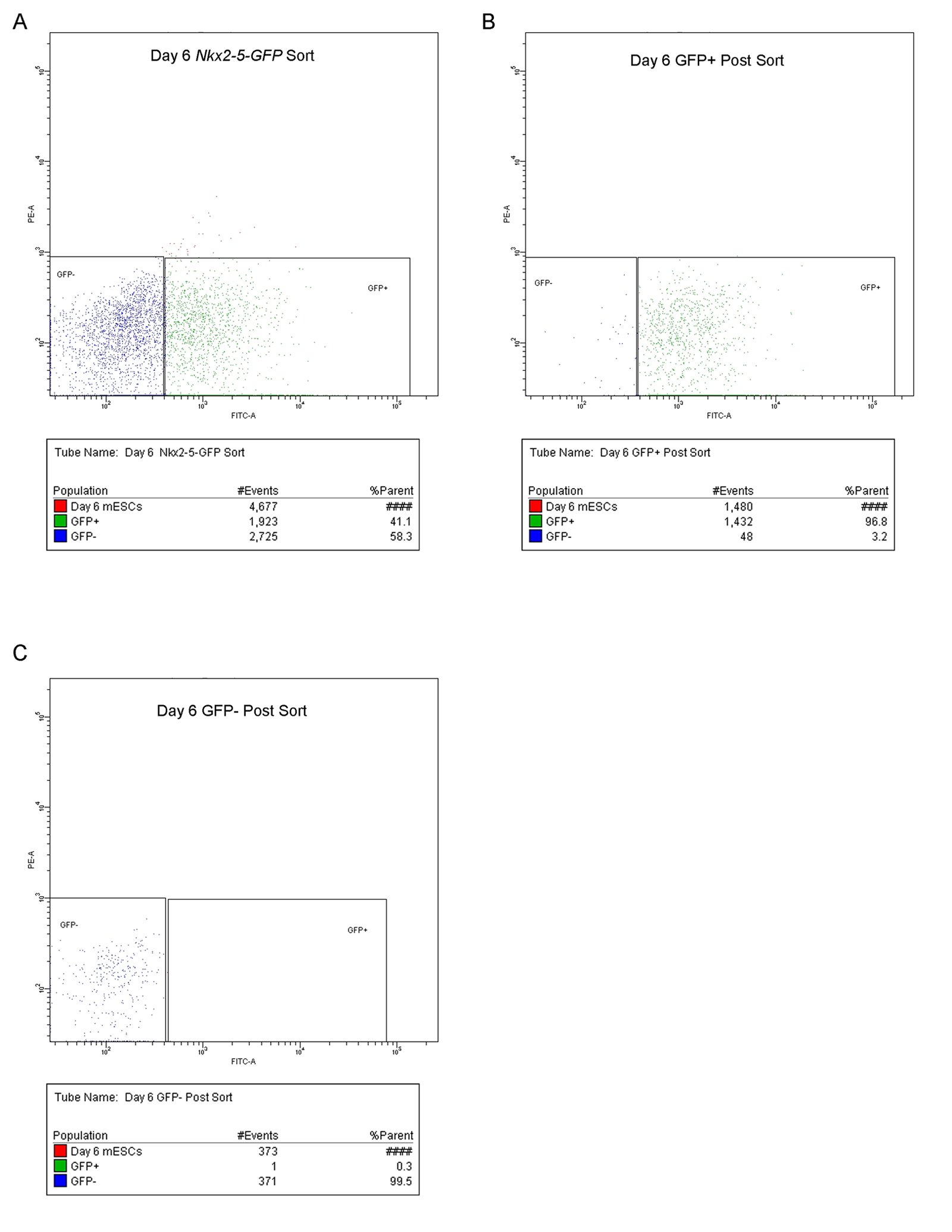

Supplement: Figure S1 — FAC Sorting Yields a Highly Enriched Population of Cardiac Fated Cells. FAC Sorting was performed on day 6 of differentiation, separating the cardiac fated cells (GFP+) from the non-cardiac fated population (GFP-) (A). The resulting samples were subjected to a second round of analyses to determine the purity of the cardiac (B) and non-cardiac (C) populations (9.95 MB TIF) [file pone.0002176.s003.tif]

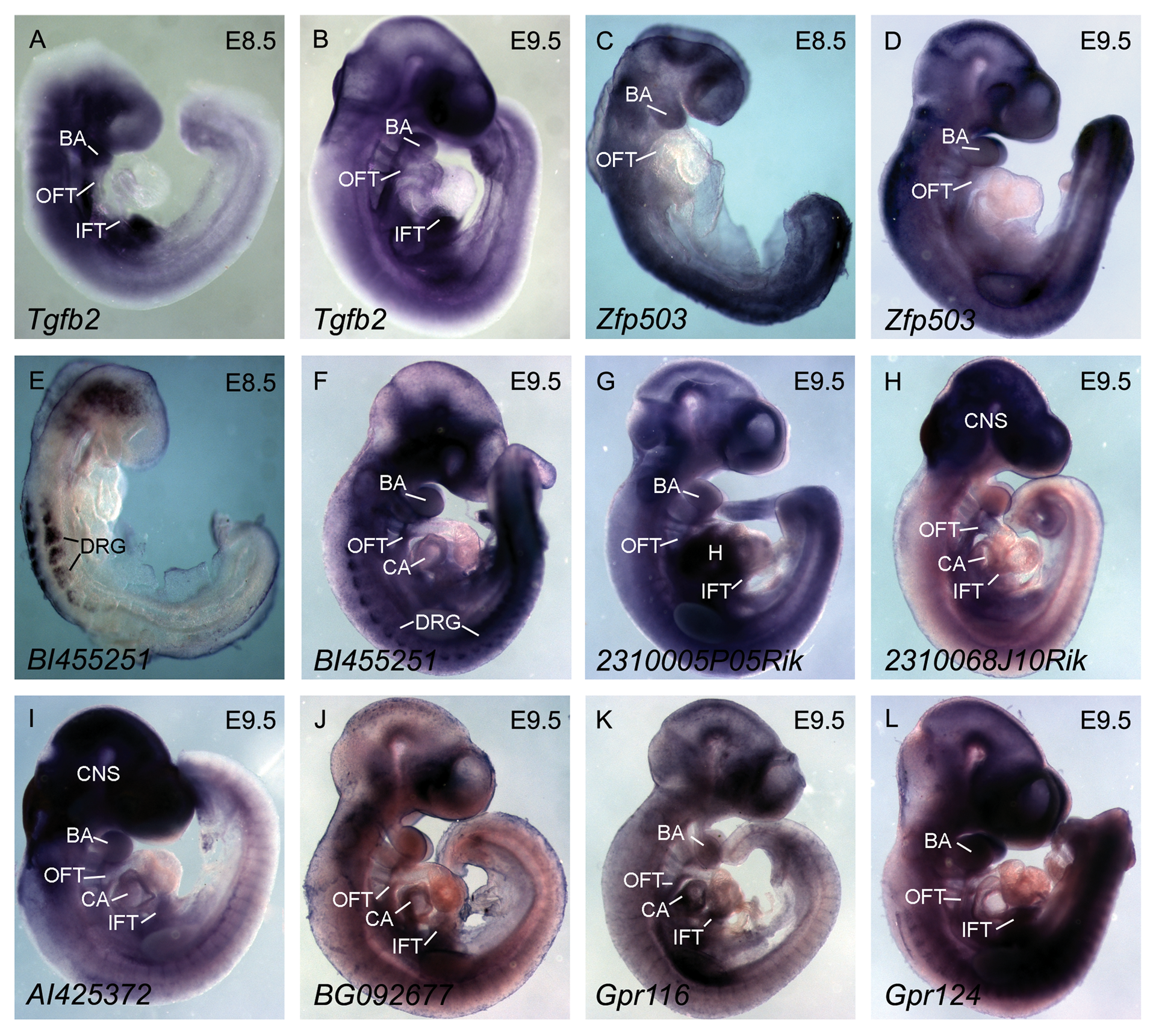

Supplement: Figure S2 — Candidate Genes Displaying Widespread Embryonic Expression in Tissues Including Key Cardiac Structures. Tgfb2 (A–B), Zfp503 (C–D), BI455251 (E–F), 2310068J10Rik (G–H) detected in cardiac structures at E8.5 and E9.5. AI425372 (I), BG092677 (J), Gpr116 (K), and Gpr124 (L) detected in cardiac structures at E9.5. H, heart; OFT, out flow tract; IFT, inflow tract; BA, branchial arches; CA, common atria; DRG, dorsal root ganglia; CNS, central nervous system. (9.93 MB TIF) [file pone.0002176.s004.tif]
